# Deep learning based Sequential model for malware analysis using Windows exe API Calls

**DOI:** 10.7717/peerj-cs.285

**Published:** 2020-07-27

**Authors:** Ferhat Ozgur Catak, Ahmet Faruk Yazı, Ogerta Elezaj, Javed Ahmed

**Affiliations:** 1 Department of Information Security and Communication Technology, NTNU Norwegian University of Science and Technology, Gjøvik, Norway; 2 Cyber Security Engineering, Istanbul Sehir University, Istanbul, Turkey; 3 TUBITAK Bilgem Cyber Security Institute, Kocaeli, Turkey

**Keywords:** Malware analysis, Sequential models, Network security, Long-short-term memory, Malware dataset

## Abstract

Malware development has seen diversity in terms of architecture and features. This advancement in the competencies of malware poses a severe threat and opens new research dimensions in malware detection. This study is focused on metamorphic malware, which is the most advanced member of the malware family. It is quite impossible for anti-virus applications using traditional signature-based methods to detect metamorphic malware, which makes it difficult to classify this type of malware accordingly. Recent research literature about malware detection and classification discusses this issue related to malware behavior. The main goal of this paper is to develop a classification method according to malware types by taking into consideration the behavior of malware. We started this research by developing a new dataset containing API calls made on the windows operating system, which represents the behavior of malicious software. The types of malicious malware included in the dataset are Adware, Backdoor, Downloader, Dropper, spyware, Trojan, Virus, and Worm. The classification method used in this study is LSTM (Long Short-Term Memory), which is a widely used classification method in sequential data. The results obtained by the classifier demonstrate accuracy up to 95% with 0.83 $F_1$-score, which is quite satisfactory. We also run our experiments with binary and multi-class malware datasets to show the classification performance of the LSTM model. Another significant contribution of this research paper is the development of a new dataset for Windows operating systems based on API calls. To the best of our knowledge, there is no such dataset available before our research. The availability of our dataset on GitHub facilitates the research community in the domain of malware detection to benefit and make a further contribution to this domain.

## Introduction

Malicious software, commonly known as malware, is any software intentionally designed to cause damage to computer systems and compromise user security. An application or code is considered malware if it secretly acts against the interests of the computer user and performs malicious activities. Malware targets various platforms such as servers, personal computers, mobile phones, and cameras to gain unauthorized access, steal personal data, and disrupt the normal function of the system. Malware has been notorious for its malicious activities and attack for decades. Malware development has become a serious activity lately as the number of target platforms increases day by day, which significantly raises the importance of developing adequate techniques to detect them. Dynamic analysis of malware in different platforms is an evolving and challenging task. According to recent statistics, 40 million new malware has been infecting systems in the first 4 months of 2018 (https://www.mcafee.com/enterprise/en-us/assets/reports/rp-quarterly-threats-jun-2018.pdf). This is evidence that malware is a significant threat in the systems and most of the time they are surpassing the capacities of malware analysts. Accordingly, great efforts are needed to protect against malware attacks.

One approach to deal with malware protection problem is by identifying the malicious software and evaluating its behavior. Usually, this problem is solved through the analysis of malware behavior. This field closely follows the model of malicious software family, which also reflects the pattern of malicious behavior. There are very few studies that have demonstrated the methods of classification according to the malware families. Proper malware labeling is a challenging issue in this domain. An anti-virus application can detect malware as a trojan, whereas, the same malware is labeled as a worm by another anti-virus application. With the advent of sophisticated malware framework, it is difficult to handle these problems. The main practical challenge faced by researchers is that malware has achieved a very complicated level of competence and effectiveness.

This allows for constant change of the code signatures (Rad, Masrom & Ibrahim, 2012). Consequently, anti-virus applications that use conventional signature-based detection methods can not detect such malware. Although a metamorphic malware that manifests itself with different code sequences in different environments, it must adopt the same behavior in all settings. Since they developed this malicious software to conduct a specific malicious activity, using this information, nearly all the methods used for the detection and classification of metamorphic malware tackle the behavioral characteristic and not the malware’s structural features. Data such as Windows API calls, DNS resolution, registry read/write operations are used in such methods to reflect malicious software behavior.

All operating system API calls made to act by any software show the overall of this program. Whether this program is alware or not can be learned by examining these actions in-depth. If it is malware, then what is its malware family. The malware-made operating system API call is a data attribute, and the sequence in which those API calls are generated is also critical the malware family Performing specific API calls is a order that represents a behavior. One of the deep learning methods LSTM (long-short term memory) has been commonly used in the processing of such time-sequential data (Shamshirband, Rabczuk & Chau, 2019).

Our research is based on the analysis of API calls made by malware on the Windows Operating System. We analyze the API calls made by different types of malware on the system to build a collection of malware-based API calls. This dataset the development of a method that can be useful for the identification of malware based on its behavior. We also construct malware detection models based on this dataset using the LSTM algorithm. This model classifies malware even though it has undergone structural changes, i.e., metamorphic malware, but in the operating system behaves like a type of malware (Shamshirband & Chronopoulos, 2019).

In our previous work, (Yazi, Catak & Gul, 2019) we applied a single layer LSTM model to detect the malware classes. In another work, (Çatak & Yazi, 2019), we describe our publicly available dataset in detail.

Our research has made the following major contribution:

• A new dataset has been developed for malware detection on Windows OS. Such a dataset does not exist in this domain.
• Malware was analyzed, and API calls were recorded by running in an isolated sandbox environment.
• Using the LSTM algorithm, which is commonly used for text classification, malware detection was modeled as a text classification problem, and the detection model for the malware type was developed.

The rest of the article is organized as follows: Section 1 briefly introduces some of the earlier works related to our problem. Section 2 describes Windows API calls, Sandbox environment and LSTM algorithm. Section 3 shows our system model. Section 4 evaluates the proposed learning model. Section 5 concludes this paper.

## Related Work

Leder, Steinbock & Martini (2009) take into consideration structural changes of metamorphic malware. Using VSA (Value Set Analysis) method, detection processes were realized by removing the unchanging code structure found in malicious software, concluding that if there is no behavioral change in samples of metamorphic malware, the detection accuracy can be 100% (Leder, Steinbock & Martini, 2009). Although they examined the metamorphic software, and they only used the operating system processes.

Vinod et al. (2010) generated malware signatures by using Windows API call sequences of metamorphic malware. The authors proposed a method for identifying and classifying malware families using these signatures. In this study, 80 malicious software has been created from each NGVCK, MPCGEN, G2, and IL_SMG families by using the VX Heavens application. A dataset is created by using software emulators in order to obtain Windows API call sequences. A dataset was created using this data. The accuracy achieved by the proposed method is 75%, 80%, 80%, and 75% for each family respectively (Vinod et al., 2010). In this study, they obtained a high detection rate. However, because they use signatures, attackers can evade this detection method.

Qiao et al. (2013) developed a detection model by considering the behavior of malware. The API call sequences of malicious software on the Windows operating system were obtained by using the Cuckoo Sandbox application. An analysis method has been developed by subtracting frequently used elements from these call sequences. The dataset used during the analyses contains 3131 malware, and the accuracy rate obtained is 94% (Qiao et al., 2013). This work is very close to our study because they use API calls. However, since they do not model the API call sequences using a sequential method, they lose this information.

Cheng, Tsai & Yang (2013) proposed an improved classification method of the behavior of malware is based on the information retrieval theory. Windows API call sequences of malware obtained from honeypots with the help of the Cuckoo Sandbox application were preprocessed with the aim to represent malicious behavior. These documents were analyzed using the TF-IDF weight, and the similarity measurement method was applied and in order to extract similarity characteristics of malicious software. A classification model was applied using these features, and the accuracy rate obtained was 97.7% (Cheng, Tsai & Yang, 2013). This work is alose close to our research because they use API calls. However, since they use TF-IDF representations of calls, Thus, they lose this information.

Mehra, Jain & Uppal (2015) proposed a method to classify 600 malware where 268 of alware were created using VX Heaven. This malicious software has been extracted from the control flow and API request graphics using the Gourmand Feature Selection algorithm to extract the required call properties. The proposed solution was implemented using the Weka tool, whereas the classification was made using histogram and Chi-square difference measurement formulas according to malware families. The classification accuracy varies between 89.00% and 99.10% for different families (Mehra, Jain & Uppal, 2015). The number of malware contained in the dataset they use is relatively low. Also, as in other studies, the API call sequence was not used.

Pirscoveanu et al. (2015) proposed a Supervised algorithm for the classification of malware, such as the Random Forests algorithm. A total of 42,000 malware behaviors were collected using the Cuckoo Sandbox application. DNS requests, Accessed Files, Mutexes and, Registry Keys data were used for the classification of Windows API calls. In addition, for the class labels of malicious software, the tags detected by Avast application are over the results provided by VirusTotal service. Trojan, Potentially Unwanted Program, Adware, and Rootkit classes are used for classification. The weighted average Area Under Curve (AUC) value of the proposed method is 0.98 (Pirscoveanu et al., 2015). The number of malware contained in the dataset they use is relatively high. Again, the API call sequence was not used.

Ahmed, Nepal & Kim proposed a different approach malware detection. The detection of malicious software in this study is not based on the characteristics of malicious software on the effects; they have on the system. So, this method does not consider the structural and behavioral features of malware that are not considered in the detection process. Still, it is limited only in the detection of abnormal behavior on the system in an ordinary situation. In this way, the authors claimed that advanced malicious software such as polymorphic, metamorphic, zero-day malware could be detected (Ahmed, Nepal & Kim, 2018).

Sami et al. (2010) developed a framework based on mining API calls of PE for malware detections. The framework includes a PE analyzer, feature generator, feature selector, and a classifier. The authors generate a set of discriminative and domain interpretable features by reading API call sets in a collection of PE files. The classifier is trained using these features. The authors also created the first public dataset and improved existing mining API methods for malware detection. The accuracy and detection rate are improved by 5.24% and 2.51%, respectively. They also reduced the false alarm rate from 19.86% to 1.51% (Sami et al., 2010). Their approach again ignores the call sequences.

Alazab, Venkataraman & Watters (2010) used a four-step methodology to extract API call features using a fully automated method. The authors disassemble, analyze, and extract the API function calls from the binary content of malware using static analysis tool IDAPro disassembler to classify program executable as malicious or benign. Statistical tests were performed on extracted calls to determine the malware class based on suspicious behavior. The sample of 386 malware used to conduct experimental tests. The authors generated six different categories of suspicious behavior of API call features based on these preliminary tests (Alazab, Venkataraman & Watters, 2010). They applied static analysis techniques to detect malware. Attackers use lots of evading techniques to bypass the analysts.

Peiravian & Zhu (2013) framework that uses permissions and API calls to detect malicious Android applications. The permissions are extracted from Android applications and combined with the API calls to characterize each application either as malware or a benign. The inherent advantage of this framework is that it does not need to involve any dynamical tracing of the system calls ut only uses simple static analysis to find system functions involved in the application. Experiments on real-world applications demonstrate the good performance of the framework for malware detection. Furthermore, the framework can be generalized to all mobile applications for malware detection (Peiravian & Zhu, 2013).

Kolosnjaji et al. (2016) proposed an approach for malware classification that uses hybrid neural networks containing two convolutional layers and one recurrent layer o obtain the best features for classification. The authors optimal classification results by combining convolutional and recurrent layers in the neural network architecture. The approach outperformed not only other simpler neural architectures, but also most widely used hidden Markov models and support vector machines (Kolosnjaji et al., 2016).

Tian et al. (2010) proposed a scalable approach for malware vs. cleanware classification and malware family classification by investigating behavioral features using logs of various API calls. The authors used an automated tool running in a virtual environment to extract API call features from executable. Later, pattern recognition algorithms and statistical methods are applied to differentiate between files. The research benefited from a dataset of 1,368 malware and 456 cleanware to conduct experimental results. As per the result, this approach provides an accuracy of over 97% distinguishes malware from cleanware. The the classification of malware into different families (Tian et al., 2010).

Alazab et al. (2010) proposed an approach to detect obfuscated malware by investigating the structural and behavioral features of API calls. The authors sed n-gram statistical analysis for API calls to analyze the similarities and distance of unknown malware with known behavior so that obfuscated malware could be detected efficiently. The authors used a dataset of 242 malware and 72 benign files to obtain experimental results. The approach demonstrates the accuracy of 96.5% for the unigram model (Alazab et al., 2010).

As far as the suggested studies are examined, it is generally seen that sequential data loss methods such as TF-IDF are preferred in the, or traditional machine learning methods are applied. Although deep learning are not algorithmically new, they have become in the field of machine learning today, as they are easy to implement technologically and can be trained with high-performance computation on systems such as GPUs. For this reason, our study differs from other studies.

## Preliminaries

In this section, we briefly introduce Windows API calls, cuckoo sandbox environment, VirusTotal service, and LSTM algorithm used for our proposed malware classification model.

### Windows API Calls

The Windows API is an interface to application developers for developing applications on the Windows operating system (Hampton, Baig & Zeadally, 2018), designed mostly for the interaction between developers and the operating system. Therefore, the operating system offers many services as API (https://docs.microsoft.com/en-us/windows/desktop/apiindex/windows-api-list).

An application developed to run on the Windows operating system must call the interfaces presented as APIs to use a function offered by the operating system. When an application is running on any operating system, it calls several API to complete an action. For example, when an application is requested to create a file, *CreateFileA* Windows API (https://docs.microsoft.com/en-us/windows/desktop/api/FileAPI/nf-fileapi-createfilea) is called. All API calls made by an application on the system can show the overall behavior of that application. Therefore, API calls-based approach is widely applied in the dynamic malware analysis showing how malware can behave accurately.

In this study, we extract Windows API calls made by malware on the operating system, and generate a feature set. Later, we use these features to train the classifier in order to detect malware.

### Cuckoo Sandbox

The Cuckoo Sandbox app is a free and public sandbox application compatible with different operating systems (Ali et al., 2019). A detailed analysis report of the files considered as suspicious (Noor, Abbas & Shahid, 2018) can be produced as part of malware analyses using this application.

With the Cuckoo Sandbox application, it is possible to prepare and run malicious software in an environment similar to a real working environment. It’s used to analyze files and collect comprehensive analysis results about the behavior and structural features of malicious software, such as API calls of malware, network traffic, memory dump, etc. The collected data are saved in a MongoDB database in JSON format.

Cuckoo Sandbox has two main components. The first component is the management machine used to start the analysis of malware, to store the results in the database, and to start the web service provided for the users. The other component is the analysis machine, virtual or physical machine, on which the malicious software is run, the actual analysis is performed, similar to the real working environment of the malicious software.

In our study, the Windows API call sequences representing the behavior of malware are collected using this application.

### VirusTotal Service

VirusTotal is an online service that analyzes suspicious files or URLs (Shiel & O’Shaughnessy, 2019). Different antivirus application engines and website browsers execute suspicious files/URLs for malicious activities. Each antivirus application engine provides a detailed report, including registry access, DNS resolutions, etc. VirusTotal service provides analysis reports from antivirus applications with an interface without any interpretation, because this service includes an extensive analysis archive, users can perform a new analysis as well as other analysis reports of other users.

VirusTotal provides an interface for receiving services without using a web browser (VirusTotal Public API v2.0), giving the possibility to application developers to nalyze files and URLs (Martn, Hernndez & de los Santos, 2019) automatically.

The body of the response is usually a JSON object containing the analysis results of antivirus application engines or web browsers separately.

We have identified the families of malware by processing the results we obtain from the API, and we have assigned labels to each malware.

### Long-Short Term Memory

LSTM is a Recurrent Neural Network (RNN) based deep learning method (Muzaffar & Afshari, 2019). LSTM was developed because RNN not successful enough in long-term learning. LSTM has an architecture that can remember and learn any long-term dependency at random intervals. It is considered a successful method to analyze data or events that have a specific relationship, especially in order of time (Hochreiter & Schmidhuber, 1997). For example, if the time series data are **x** = {*x*_1_, …, *x*_*T*_}, **h** = {*h*_1_, …, *h*_*T*_} is the hidden vector sequence and **y** = {*y*_1_, …, *y*_*T*_} shows an output vector sequence, then *T* iteration is defined as follows:

(1) \begin{eqnarray*}{h}_{t}=& \mathcal{H}({W}_{xh}{x}_{t}+{W}_{hh}{h}_{t-1}+{b}_{h})\end{eqnarray*}

\begin{eqnarray*}{y}_{t}=& \mathcal{F}({W}_{hy}{h}_{t}+{b}_{y}) \end{eqnarray*}

where *W*_*xh*_, *W*_*hh*_, and *W*_*hy*_ are the computation time connection weight matrices and $\mathcal{F}$ is the activation function.

### System architecture

This research has two main objectives; first, we created a relevant dataset, and then, using this dataset, we did a comparative study using various machine learning to detect and classify malware automatically based on their types.

### Dataset creation

One of the most important contributions of this work is the new Windows PE Malware API sequence dataset, which contains malware analysis information. are 7107 malware from different classes in this dataset. The Cuckoo Sandbox application, as explained above, is used to obtain the Windows API call sequences of malicious software, and VirusTotal Service is used to detect the classes of malware.

**Figure 1:**
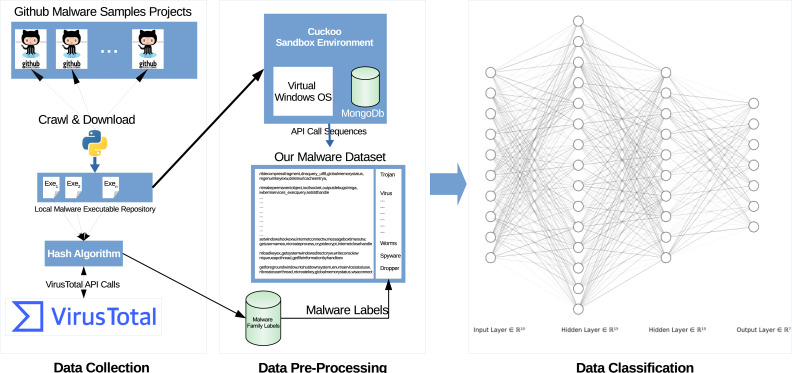
General system architecture. Architecture consists of 3 parts; data collection, Data pre-processing, and Data classification.

Figure 1 illustrates the system architecture used to collect the data and to classify them using LSTM algorithms.

Our system consists of three main parts, data collection, data pre-processing and analyses, and data classification.

The following steps were followed when creating the dataset.

Cuckoo Sandbox application is installed on a computer running Ubuntu Linux distribution. The analysis machine was run as a virtual server to run and analyze malware. The Windows operating system is installed on this server. The firewall has been turned off, and no operating system updates have been made to prevent any malware from running. The tools versions are; *Ubuntu* 17.10, *Cuckoo* 2.0.4, Windows 7 *Analysis OS*, *VirtualBox* 5.1.30, *Python* 3.6.5.

**Figure 2:**
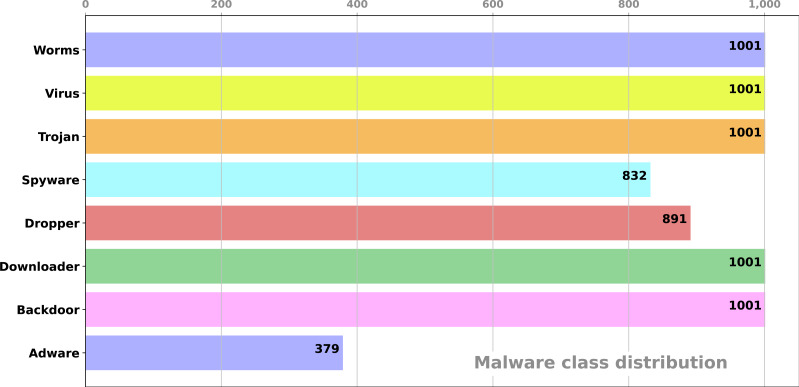
The number of malware in each class.

Once the data are collected, we start the process to analyses and pre-process the malware using Cuckoo as a dynamic More than 20,000 malware have been run separately in the Cuckoo Sandbox application, and the results are all stored in a MongoDB database. From this analysis information, we obtained the behavioral data of the malware on the analysis machine. This behavior is all Windows API call requests that the malware has made on the Windows 7 operating system. Some of the data pre-processing activities are:

• **Indexing Windows API calls**: When we examined the Windows API calls in the dataset, we found that there were 342 different API calls. These API calls are indexed from 0 to 341. As a result, each row in the dataset represents the API call sequence for alware analysis.
• **Dataset filtering**: Since these malware are developed for a specific application with a target, we filtered the API call sequence and discarded the rows that did not contain at least 10 different API calls from the dataset.
• **Analysis of malware using VirusTotal Public API**: We determined the hash values of each of malware that we analyzed and inquired these hash values with VirusTotal service. We stored the analysis results from the VirusTotal service into a database. Thus, each malware was analyzed by many different antivirus engines, and the results of the analysis were recorded.
• **Processing of analysis results**: Based on the results of each analysis, we have obtained using this service, we have labeled all the malware. During this process, we found out that different antivirus applications give different results for the same malware, or sometimes not every antivirus application can detect every malware. For example, when we analyze the hash value of 06*e*76*cf*96*c*7*c*7*a*3*a*138324516*af*9*fce*8 in the VirusTotal service, many of the software indicate that this file is a worm, while *DrWeb* says it is a trojan, and *Babable* end that it is a clean file. Therefore, in determining the classes of malware, we considered the majority class in the analyzes. If the majority of engines agree that a particular sample is malicious, then it is as positive.
• **Creating the dataset**: Finally, the labeled training dataset was created by matching the Windows API call sequences and the malware classes. This dataset contains 8 different classes of malware. Our dataset is publicly available on GitHub website (https://github.com/ocatak/malware_api_class). The number of malware included in these classes is shown in Fig. 2.

In this study, the malware classification method was developed by using the LSTM algorithm. The LSTM algorithm does not require any vectorization model, such as TF-IDF, because it works with sequential data. However, it is necessary to compare the classification performance of the developed method with other traditional machine learning algorithms such as support vector machine, decision tree, k-nearest neighbor. TF-IDF model traditional classification algorithms

In ‘TF-IDF’, we describe how to transform the text with the TF-IDF method.

### TF-IDF

Term frequency-inverse document frequency based text dataset vectorization is a traditional method to create a numerical dataset. Each term *t* in a document *d* is assigned a weight assignment according to the frequency of occurrence in that document (Manning, Raghavan & Schütze, 2008). This process is called term frequency, *tf*_(*t*,*d*)_. The vector, thus formed, can be considered as a digitized summary of a document. Let the term *t* be represented by *f*(*t*, *d*) of the raw frequency. Term frequency can be found as follow:

(2) \begin{eqnarray*}t{f}_{(t,d)}=f(t,d)\end{eqnarray*}

However, a dataset generated only by the term frequencies assigns equal importance to each term. The of occurrence of any in a collection The inverse document frequency (IDF) is defined as the logarithm of the division of the number of documents in the, *N*, to the document frequency, *df*_*t*_.

(3) \begin{eqnarray*}id{f}_{t}=\log \nolimits \frac{N}{d{f}_{t}} \end{eqnarray*}

Thus, if the *df*_*t*_ in the documents in the collection is low, the *idf*_*t*_ value will be high, and in the high *dt*_*f*_ frequency the *idf*_*t*_ value will be low. To calculate the weights of the terms contained in each document, the term frequency, *tf*_*t*,*d*_, and inverse document frequency, *idf*_*t*_, are combined to form the term frequency-inverse document frequency (TF-IDF) matrix.

(4) \begin{eqnarray*}tf-id{f}_{t}=t{f}_{t,d}\times id{f}_{t}\end{eqnarray*}

In this study, the malware classification method was developed by using the LSTM algorithm. The LSTM algorithm does not require any vectorization model, such as TF-IDF, because it works with sequential data. However, it is necessary to compare the classification performance of the developed method with other traditional machine learning algorithms such as support vector machine, decision tree, k-nearest neighbor. The TF-IDF model is used only because classification algorithms work with numerical data only. TF-IDF is not used in our odel.

### Classifier learning

The steps 1–7 are run separately for each type of malware, creating classification models for 8 different types. The experiments are done using the Python programming language and machine learning libraries Keras, Tensorflow, and Scikit-learn. We used the Keras library to build LSTM networks. We have created a two-tier LSTM structure.

In Algorithm 1, we explain the general steps of our classifier building stage. Accordingly, our algorithm’s both time and space complexity is O(n). In the algorithm, there is the main loop to build a representative classifier for each distinct label.

 
____________________________________________________________________________ 
Algorithm 1 Classifier learning steps 
__________________________________________________________________________ 
  1:  Inputs: 
        Malware API call dataset X, malware class labels Y , class 
          set C 
 2:  for each c ∈ C do 
 3:          ⊳ Labels that are belongs to c will be 1, rest are 0 for binary classifier 
  4:       Xtrainc,Xtestc,Ytrainc,Ytrainc ← train_test_split(X,Yc,0.8)          ⊳ 0.8 
     training data, 0.2 validation data 
  5:       hc ← LSTM_model_fit(Xtrainc,Ytrainc)  ⊳ LSTM model building with 
     train dataset 
  6:  end for 
 7:  Outputs: 
        A set of classifiers set for each class C, h : X ↦→ Y________________________    

**Figure 3:**
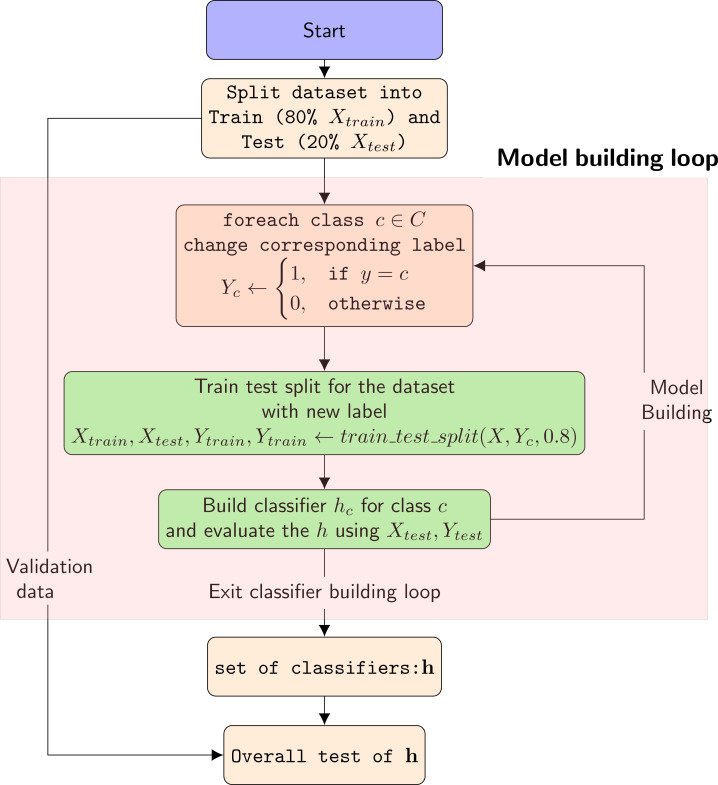
FLFlow chart of the overall system.

Figure 3 shows the flowchart of the overall method. The process of malware classification includes the following steps in the proposed solution:

1. We select the that we want to classify.
2. We process the dataset for the selected malware type. The model assigns label 1 to the malware type information of interest and label 0 to other categories.
3. A two-tier LSTM-based classification model is defined and created.
4. The classifier is trained using 80% of the data during the training phase. Validation is performed during training with 20% of the data allocated for training.
5. The trained classifier is tested using 20% of the data in the dataset. During this process, API calls of a new software are shown to the classifier and a class label is assigned to these new instances based on the voting results of the models.
6. Training and test results are recorded.
7. The classifier is trained using 80% of the data during the training phase. Validation is performed during training with 20% of the data allocated for training.

**Figure 4:**
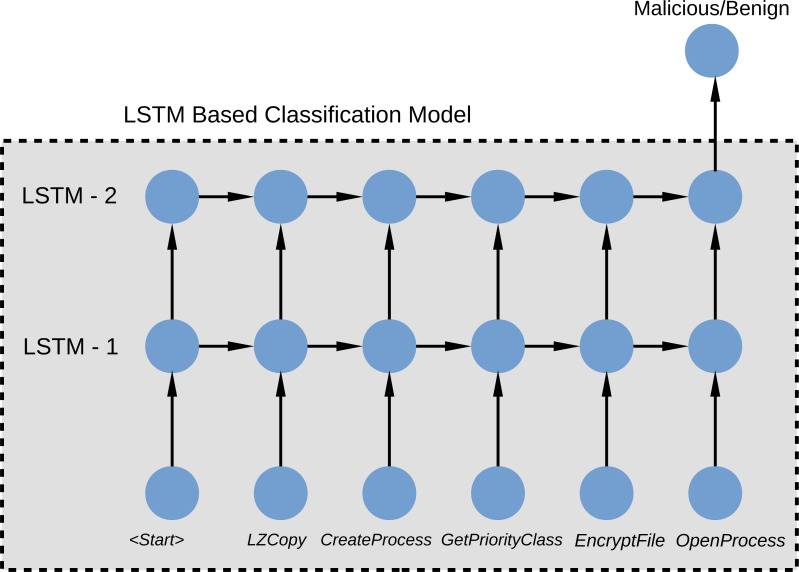
LSTM classification model with Windows API calls.

The training process is shown in Fig. 4. The LSTM network model receives the API calls that each malware makes on the Windows operating system and assigns the class label to $\hat {y}$. As we apply the classifiers to each of the 8 malware classes, our classifier is binary ones. *Log loss* function is used as the loss function, shown in Eq. (5).

(5) \begin{eqnarray*}l(\mathbf{y},\hat {\mathbf{y}})= \frac{1}{L} \sum _{l=1}^{l={|}L{|}}- \left( {y}_{l}\log \nolimits ({\hat {y}}_{l})+(1-{y}_{l})log(1-{\hat {y}}_{l}) \right) \end{eqnarray*}

### Evaluation

Since the dataset that is used in our experiments highly imbalanced, traditional accuracy based performance evaluation is not enough to find out an optimal classifier. We used four different metrics, the overall prediction accuracy, average recall, average precision (Turpin & Scholer, 2006) and *F*1 score, to evaluate the classification accuracy, which measurement metrics in information retrieval (Manning, Raghavan & Schütze, 2008; Makhoul et al., 1999).

## Experiments and Results

In this study, we performed the classification of malware belonging to different families in the dataset described in Section 3.1 with the LSTM algorithm. All experiments were run in a Python environment, and all algorithm codes have been modified to build a classification model from the given data. Our codes can be accessed on our GitHub repository (https://github.com/ocatak/malware_api_class/tree/master/src).

As we have explained in the previous sections, we can analyze the sequential data with the LSTM algorithm that we have used within the scope of this study and create a classification model as a result. When it is desired to a model using other classification algorithms, the existing text data should be digitized. For this purpose, we used the TF-IDF method, which is the most common text digitization method. We used our numerical data set with this method with the k-Nearest Neighborhood, Decision Tree (DT), and Support Vector Machine (SVM) algorithms. In ‘TF-IDF’, we describe how to transform the text with the TF-IDF method.

In this section, we will share the experimental results for binary and multi-class classification separately. We designed 2 different experiments for multi-class classification; single layer LSTM and two layers LSTM. The purpose of doing this is that two-layer LSTM models are overfitting in some cases.

## Model configuration

Since we wanted to create a model for each class, we edited the class information in the dataset. We assigned 1 for the malware class to be analyzed and 0 for the others. Since we do this while creating each classification model, these models perform binary type classification. For example, the result of the classification model created for the Adware class is *Adware* or *other*.

**Figure 5:**
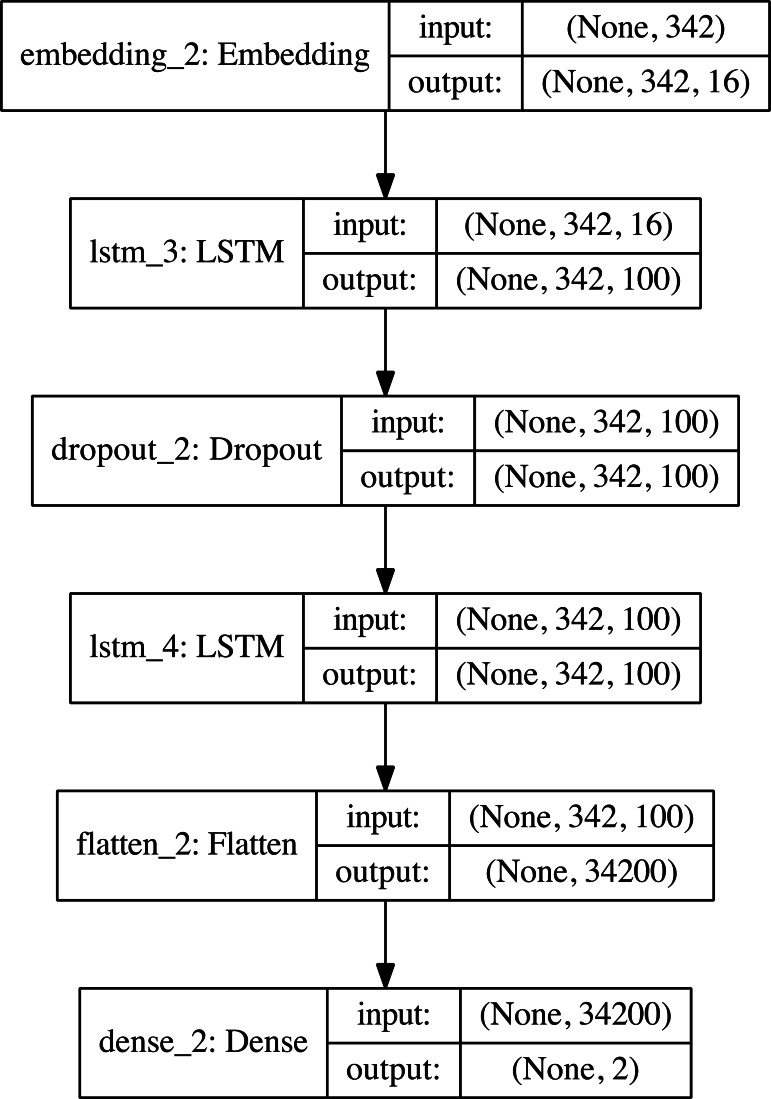
The classification model layers and their neurons.

We used *tanh*, *relu*, *sigmoid*, *softplus*, *softsign*, *softmax* and *linear* activation functions and created eight different models for each malware class. We have used the same flow layers, although there are different numbers of data analyzed at the stage of creating malware models. An example of the flow layers of the models is the Adware classification model, which is shown in Fig. 5.

## Binary classification results

Table 1 shows the classification performance of LSTM and conventional algorithms results. Although the accuracy rates of the traditional methods and the deep learning methods seem to be similar, F1 values give more meaningful information due to the lack of close class distributions. F1 represents the balance between precision and recall values. Therefore, the F1 values obtained by the deep learning method better than the F1 values obtained by the traditional methods. This suggests that deep learning is better for analyzing malware behavior.

**Table 1:** LSTM and conventional algorithms classification results.

	**Adware**	**Backdoor**	**Downloader**	**Dropper**	**Spyware**	**Trojan**	**Virus**	**Worm**
**LSTM**								
Accuracy	0.985	0.877	0.909	0.877	0.876	0.835	0.927	0.865
Precision	0.90	0.60	0.72	0.52	0.46	0.36	0.76	0.57
Recall	0.77	0.40	0.57	0.44	0.42	0.22	0.70	0.37
F1	**0.83**	**0.48**	**0.64**	**0.48**	**0.44**	**0.27**	**0.73**	**0.45**
Func.	tanh	tanh	soft sign	soft sign	soft sign	soft sign	tanh	soft sign
								
**k-NN**								
Accuracy	0.976	0.834	0.870	0.819	0.879	0.841	0.859	0.823
Precision	0.89	0.38	0.62	0.19	0.45	0.33	0.47	0.36
Recall	0.59	0.26	0.20	0.13	0.26	0.12	0.21	0.23
F1	0.71	0.31	0.30	0.16	0.33	0.18	0.29	0.28
								
**DT**								
Accuracy	0.954	0.824	0.839	0.815	0.824	0.786	0.856	0.825
Precision	0.53	0.39	0.43	0.32	0.30	0.26	0.48	0.41
Recall	0.80	0.41	0.48	0.38	0.41	0.28	0.52	0.40
F1	0.64	0.40	0.45	0.35	0.35	**0.27**	0.50	0.40
								
**RBF SVM**								
Accuracy	0.979	0.875	0.876	0.881	0.899	0.867	0.888	0.875
Precision	1.00	0.91	1.00	0.84	0.79	0.87	1.00	0.93
Recall	0.59	0.14	0.12	0.09	0.16	0.07	0.18	0.18
F1	0.74	0.25	0.22	0.16	0.27	0.12	0.31	0.29
								
**Sigmoid SVM**								
Accuracy	0.950	0.864	0.860	0.872	0.886	0.859	0.862	0.851
Precision	0	0.87	0	0	0	0	0	0
Recall	0	0.06	0	0	0	0	0	0
F1	0	0.12	0	0	0	0	0	0

**Notes.**

**Bold values indicate the best value of each column.**

As expected, based on the experimental results, the classification performance of the models created by using traditional classification algorithms using the TF-IDF based vectorization method, where the sequence information is not used, is lower than the performance of the model created using the LSTM algorithm. Considering all four classification evaluation metrics, we proposed a practical implementation of the malware detection model using the LSTM algorithm. Our proposed method is more efficient as we obtained better scores on all the evaluation metrics.

Based on the results and the classification performance shown in Table 1, we conclude that the LSTM model is the best approach that provides the best performance for all evaluation metrics.

## Multi-class classification results

Using our data set, we created several multi-class classification models with 8 different classes are also created. Especially by using different hyper-parameters, the most successful models were tried to be obtained. *F*_1_ metric value will be used to compare the analysis results. The *classification_report* method in the *sklearn.metrics* the end library was used to calculate the *F*_1_, precision, recall, and average values of these values. The average *F*_1_ value produced by this method takes into account the precision and recall values and class weights in the dataset.

### Single layer LSTM results

**Figure 6:**
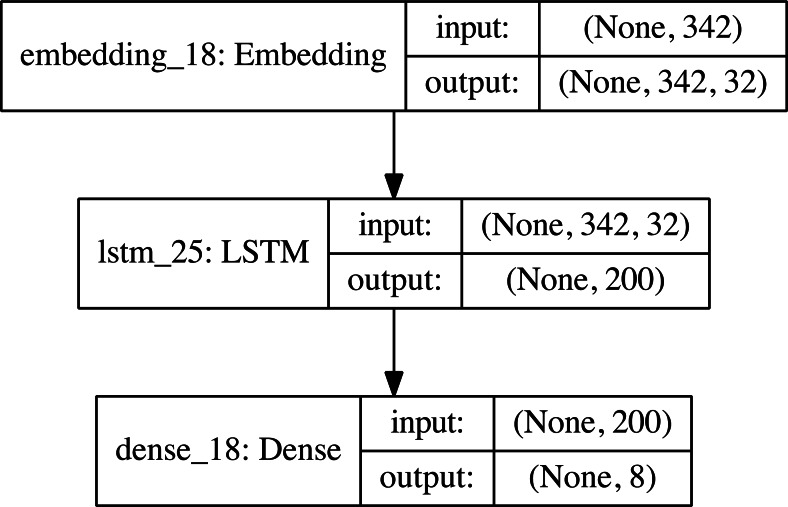
Single layer LSTM classification model structure.

Single-layer LSTM models have been created that can classify 8 different types of malware. These models produce an output between 0–7. These values represent malware class. Figure 6 shows our single layer LSTM architecture.

While creating the models, it is aimed to obtain the best classification model by using many different hyper-parameters. Table 2 shows our hyper-parameter search space.

Table 3 shows the best classification performance obtained using following hyper-parameters.

**Figure 7:**
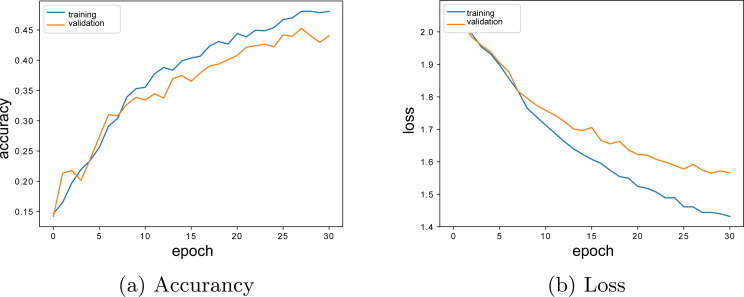
Single layer LSTM model accuracy-loss graphics. (A) Accuracy; (B) Loss.

The training history, accuracy and loss graphs of the model created by using the best hyper-parameters are given in Figs. 7A and 7B respectively. When the graphics are examined, it is seen that the model education process stops at the 30th epoch. The reason for this situation is that the model comes to the overfitting point. During the model trainings, using the *EarlyStopping* parameter, it was ensured that the education was terminated without reaching the extreme fit of the model.

The Confusion Matrix information obtained as a result of testing the trained classification model is given in Table 4.

The analysis results obtained by testing the trained classification model are shown in Table 5.

**Table 2:** Hyper-parameter search space to tune the model.

**Hyper-Parameter**	**Search Space**
Embedding layer number of units	5 - 300
LSTM layer number of units	1 - 300 Activation function: anh
Activation function	tanh , relu, sigmoid, softplus, softsign, softmax, linear kernel initializer:
kernel initializer	random_uniform, glorot_uniform, lecun_uniform, uniform Dropout:
Dropout	0.1 - 0.9 optimizer:
optimizer	adam, adadelta, adamax, nadam

**Table 3:** Best hyper-parameters.

**Hyper-Parameter**	**Value**
Embedding units	32 units:
Units	200
Activation	sigmoid
Kernel_initializer	glorot_uniform
Dropout	0.2
Optimizer	adam

### Two layers LSTM results

Two-layer LSTM models have been created that can classify 8 different types of malware. These models produce an output between 0–7.

Table 6 shows the best classification performance hyper-parameters.

**Figure 8:**
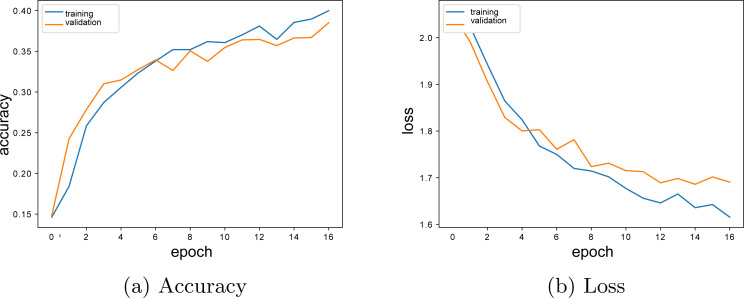
Two layers LSTM model accuracy-loss graphics. (A) Accuracy; (B) Loss.

According to Fig. 8, the model training process stops at the 16th epoch because of overfitting limit. During the model trainings, the training was ended before the model reached the extreme fit state by using the *EarlyStopping* parameter.

Confusion Matrix is given in Table 7.

Table 8 shows the two layers LSTM model classification performance results.

Multi-class classification model results obtained using different algorithms are given in Table 9.

**Table 4:** Single Layer LSTM Model Confusion Matrix.

49	0	8	4	1	5	4	0
1	92	6	15	22	44	6	17
6	10	124	22	6	22	4	5
3	12	6	80	21	53	6	0
2	22	7	16	41	48	17	9
3	23	13	25	25	90	15	6
7	6	1	19	8	20	131	3
2	30	6	17	19	62	20	55

**Table 5:** Single Layer LSTM Model Classification Results.

	Precision	Recall	*F*_1_
Adware	0.67	0.69	0.68
Backdoor	0.47	0.45	0.46
Downloader	0.73	0.62	0.67
Dropper	0.40	0.44	0.42
Spyware	0.29	0.25	0.27
Trojan	0.26	0.45	0.33
Virus	0.65	0.67	0.66
Worm	0.58	0.26	0.36
**Average**	0.50	0.47	0.47

**Table 6:** Hyper-parameter search space to tune the model.

**Hyper-Parameter**	**Search Space**
Embedding units	16 units:
units	25
LSTM-1 activation : softsign	softsign
LSTM-1 kernel_initializer	glorot_uniform
LSTM-2 activation : softsign	softsign
LSTM-2 kernel_initializer	glorot_uniform
LSTM-2 recurrent_dropout	0.2
Dense kernel_regularizer	regularizers.l2(0.01)
Dense activity_regularizer	regularizers.l1(0.01) optimizer: adam
Optimizer	adam

**Table 7:**
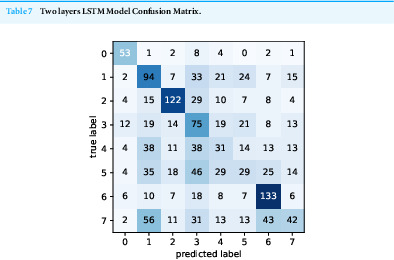
Two layers LSTM Model Confusion Matrix.

**Table 8:** Two layers LSTM model classification performance results.

	Precision	Recall	*F*_1_
Adware	0.61	0.75	0.67
Backdoor	0.35	0.46	0.40
Downloader	0.64	0.61	0.62
Dropper	0.27	0.41	0.33
Spyware	0.23	0.19	0.21
Trojan	0.25	0.14	0.18
Virus	0.56	0.68	0.61
Worm	0.39	0.20	0.26
**Average**	0.40	0.41	0.39

**Table 9:** Multi-class classificaton model performance results.

	Precision	Recall	*F*_1_
LSTM	0.50	**0.47**	**0.47**
2LSTM	0.40	0.41	0.39
DT	0.40	0.41	0.40
kNN	0.35	0.35	0.34
RF	0.46	**0.47**	0.46
SVM	**0.78**	0.29	0.29

## Results

As expected, based on the experimental results, LSTM based malware classification than the TF-IDF based conventional machine learning algorithms’ classification performance. Considering all the four classification evaluation metrics, we proposed a practical implementation of the malware classification model using sequential based Windows OS API calls and LSTM networks. Our proposed method is more efficient as we get better scores on evaluation metrics.

Based on the results presented in Section 4.2 and Section 4.3 the computation results shown in Table 1, it can be concluded that LSTM is the best approach that provides best results for all evaluation metrics.

On the other hand, if we compare the training periods, the training of LSTM models takes longer. The features of the computer where the experiments are carried out are as follows; Windows 7(64 bit), Intel(R) Core(TM) i7-2600 CPU@ 3.40GHZ, and 6GB RAM. The training durations are

• Single layer LSTM: 64.78 min
• Two lyers LSTM: 42.82 min
• Decision Tree: 1.36 min
• kNN: 5.26 min
• RF: 6.8 min
• SVM: 13.62 min

Sequential data are used for LSTM While making the vectorizing process using the TF-IDF model with conventional methods.

According to confusion matrices on Tables 4–7, we can conclude that the most discriminating malware class is *Trojan* and the least discriminating class is *Spyware*. From these results, we can conclude that the samples belonging to the Spyware malware class do not follow a particular API call sequence. Although the model complexity was increased with 2 layers of LSTM, and we did not see a significant difference when we examined the classification performances.

## Conclusion and Future Works

The purpose of this study was to create a dataset by obtaining runtime system calls made by 7107 malicious software on Windows 7. As a result, we built a dataset that contains the malware behavioral data at runtime and class labels to which the software was included. classification model is proposed, and this dataset created a model for malware detection using deep learning method LSTM.

We build separate classification models for each malware family and found that the results of the classification of these models showed a success rate between 83.5% to 98.5%. We can say that our classification method exhibits excellent performance because VirusTotal services malware family labeling cannot be accurate. Also we showed that single-layer LSTM and two-tier LSTM models achieved almost the same classification results. Thus, the complexity of the model doesn’t increase performance. Although our dataset contains instances that belong to some malware families with unbalanced distribution, we have shown that this problem does not affect classification performance.

Our research can be applied to other malware families as well because the behavior demonstrated by metamorphic malware in an operating system is similar do the other family members. As a result, the LSTM approach can be used in the classification of metamorphic malware, and this study is a conceptual proof of this finding.

It is assumed that the malware did not detect the sandbox environment in the dataset we used in this analysis. Some sophisticated malware the potential to identify that they are being run in an isolated environment by using the images methods have started to be introduced instead of running such malware in a sandbox environment to detect such malware that changes behavior by detecting an anti-VM environment. As future work, we intend to use malware images method to classify the correctly labeled dataset. Besides, we want to use other sequential data classification algorithms used before deep learning.

## Additional Information and Declarations

### Data Availability

The following information was supplied regarding data availability: The Mal-API-2019 Dataset is available at GitHub:
https://github.com/ocatak/malware_api_class
Python source folder is also available at GitHub: https://github.com/ocatak/malware_api_class/tree/master/src.
